# Material Removal Modeling for Free-Form Rubber Materials

**DOI:** 10.3390/ma18071584

**Published:** 2025-04-01

**Authors:** Yaodong Zhang, Weiqi Fu, Yanzhao Ma, Xiang Chai, Jiong Bai, Zhiqiang Zhang

**Affiliations:** School of Power and Mechanical Engineering, Wuhan University, Wuhan 430072, China; 2018302080378@whu.edu.cn (Y.Z.); 2022282080090@whu.edu.cn (W.F.); mayanzhao@whu.edu.cn (Y.M.); 2019302080371@whu.edu.cn (X.C.); 2019302080325@whu.edu.cn (J.B.)

**Keywords:** robotic grinding, material removal modeling, rubber grinding, free-form workpiece

## Abstract

Manual grinding presents significant challenges for the task of high-precision shallow uniform grinding of curved rubber materials. The use of robots and grinding discs has the potential to substantially improve both the precision and efficiency of grinding tasks. To achieve this, a precise material removal model is essential to facilitate the optimization of robot grinding parameters and path planning. Nevertheless, most existing models primarily focus on metal materials or deep grinding depths, rendering them inapplicable to rubber materials. Therefore, this study investigates the contact mechanics during disc grinding of rubber materials and quantifies the distributions of grinding pressure and speed. Additionally, it evaluates the effects of different elastic deformation and wear stages on the grinding process, ultimately developing a material removal model for free-form rubber surfaces based on the Preston equation. The validity of the model is experimentally verified. Furthermore, the study shows that, when processing workpieces with different surface shapes, the study indicates a negative correlation between both the grinding width and the grinding pressure with the curvature radius.

## 1. Introduction

**Figure 1:**
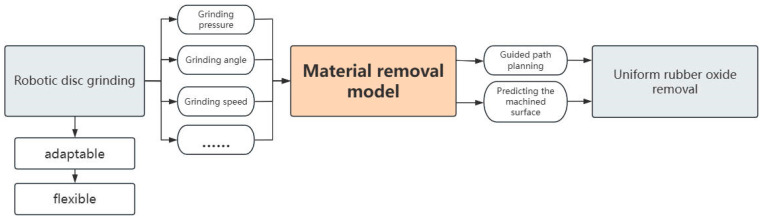
Significance of material removal modeling.

The rubber material has good thermal insulation, mechanical properties, and remarkable elasticity, enabling its application to objects of diverse shapes and sizes [1]. This material is characterized by ease of installation and use. In the aerospace industry, it is suitable to be utilized as an insulating layer within a solid rocket motor combustion chamber, protecting against high temperatures and pressures. However, it generates a smooth oxide layer on the surface after internal vulcanization, which needs to be ground to complete the surface roughening before use. Conventionally, the grinding of rubber materials has been performed manually, but this method has been shown to lack stability and result in inconsistent outcomes [2]. Robotic grinding is considered a good alternative for its flexibility and stability. To grind curved workpieces, a flexible disc grinder is usually installed at the end of the robot. Disc grinding is a widely used machining technique, particularly effective for processing materials with complex curved geometries [3,4]. The flexible layer of the grinding disc adapts to the shape of the material surface to achieve a large contact area and improve processing efficiency, and it mainly grinds the surface of the workpiece, allowing better control of the depth of material removal and the quality of the ground surface. Accurate material removal modeling enables reliable prediction of the surface of the processed material while helping to complete the grinding path planning, as shown in Figure 1. However, the coupled effects of grinding pressure, grinding speed, feed rate, material shape, and grinding path present significant challenges in modeling the material removal depth contour (MRDC) [5]. An efficient and reliable material removal model for rubber materials is needed to perform more accurate grinding of rubber materials.

The rubber grinding mechanism differs from the metal modeling mechanism. For Nitrile Butadiene Rubber (NBR), the main form of wear is abrasive wear at grits greater than 180 grit [6]. Rubber material has high elasticity and low hardness, and the contact situation with flexible grinding discs is also different from that of metal materials, and the deformation of the material itself cannot be neglected when the Young’s modulus of rubber material is low. For the super soft rubber elastomers with Young’s modulus of 0.186 MPa, Poisson’s ratio of 0.2, and hardness of 10 HA, Xu proposed a grinding removal model for dual flexible materials and analyzed the influence of different thicknesses of planar workpieces under different grinding pressures and grinding angles [7].

The key to the grinding disc material removal model is the contact force model while clarifying the distribution of contact pressure [8]. The Hertzian model is the most classical and widely used contact force model [9]. However, this model assumes an elliptical contact area, which does not hold when grinding with a disc, as the actual contact area is substantial and non-elliptical, making it incompatible with the Hertzian assumptions [10]. For modeling the contact forces for disc grinding, H. Nasri assumed that the disc is locally rigid and used a cantilever beam model to calculate the grinding forces [11]; B.J. Ulrich investigated the contact forces of grinding with a rigid tilting disc and found that the material removal and grinding forces were mainly caused by the dynamic cutting forces of the material [12]. Considering curved workpieces, Feng examined the relationship between contact force and downward pressure depth at a fixed angle, finding that the contact force is quadratically related to the depth of contact [13]. Alternatively, the Finite Element Method (FEM) is also convenient for contact force analysis, but the process is time-consuming and costly [14,15].

Secondly, most scholars have focused on analyzing the relationship between the material removal model and various complex influencing factors. Li analyzed the grinding forces of slip friction, plowing, and cutting effects in three wear stages and proposed a material removal rate (MRR) prediction model based on individual abrasive grains [16]; Yang held a similar viewpoint and proposed a kinematic trajectory material removal model based on individual spherical abrasive grains [17]; Huan analyzed the contact calculation in the flexible grinding process (FGP) and developed a methodology to convert a non-constant contact model process into a constant contact grinding process, based on which a material removal rate (MR) model was developed [18]; Zhou proposed an elastic extrusion model for analyzing the contact pressure distribution between a flexible tool and a hard and complex surface, and proposed a dynamic local projection algorithm for efficient and accurate MRD calculations [19]. Lv uses Preston’s equation to calculate the material removal rate in the region of nonlinear pressure distribution [20]. Pan collected a large amount of grinding data based on the use of a black box model with grinding parameters as inputs and the material removal rate as outputs [21]; Wang employed the Cabaravdic model to investigate the relationship between material removal rate and grinding parameters, using experimental fitting to determine the required coefficients [22].

However, the modeling approach in the current study fails to consider the grinding of curved rubber materials, which involves a relatively larger grinding contact area. Xiao proposed a grinding disc contact force model for free-form surfaces, which achieved uniform material removal considering the variation of workpiece curvature. However, this model was primarily applicable to scenarios with small contact areas and exhibited significant deviation when the tilting angle was small and the depth of contact was large [23]. Wang proposed a contact pressure analysis method based on the shape of the tilted disc contact area with a curved surface, which used the geometric method for quick analysis. Still, the analysis is not accurate enough considering only the case where the curvature of the surface does not change much [24]. Previous studies indicate insufficient research on rubber materials requiring only surface oxidized layer removal by grinding (depth < 0.3 mm). Particularly for high-hardness rubbers (HA > 60), there is still a lack of an accurate shallow material removal model. This paper therefore proposes a novel material removal model for shallow-layer grinding using inclined grinding discs on free-form rubber surfaces.

## 2. Modeling of Rubber Material Removal

The present study investigates the rubber material utilized in the inner wall of the combustion chamber of a rocket engine, which possesses a Poisson’s ratio of 0.49 and a hardness that exceeds 69 HA.

This section will focus on modeling the contact between the flexible grinding disc and the free-form material. The material removal depth model is obtained by associating the resulting model with the Preston equation.

### 2.1. Analysis of Grinding Mechanisms

**Figure 2:**
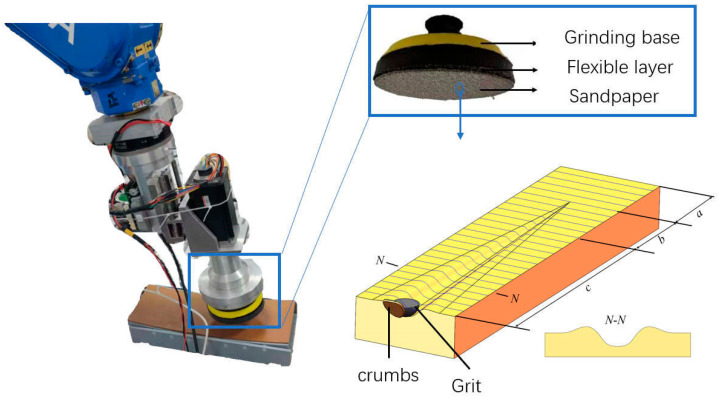
Schematic diagram of the grinding process. (**a**) Sliding, (**b**) Plowing, (**c**) Abrasive Wear.

The disc grinding device uses a flexible disc, as shown in Figure 2, which incorporates sponge pads in the sandpaper and the grinding base. These pads exhibit strong adhesion to the workpiece surface during the grinding process, facilitating efficient passage when encountering the curved insulation layer, thereby enabling effective grinding.

In general terms, the disc’s surface is employed to make contact with the adiabatic layer, thereby generating a certain degree of pressure and relative sliding, thus facilitating the removal of material from the surface of the adiabatic layer. When the abrasive grain is larger than 70 um and the sandpaper is smaller than 180 grit, the form of wear changes from adhesive wear to abrasive wear [6]. Abrasive wear is classified according to the depth of penetration and consists of three stages—sliding, plowing, and cutting—as illustrated in Figure 2 [25]. The grinding process can be categorized according to the deformation behavior of the material. The deformation behavior can be divided into two primary stages—elastic deformation and plastic deformation. The transition between these stages occurs when the contact pressure and the relative sliding velocity exceed a specific critical threshold. It has been determined through experimental analysis that, under higher friction conditions and greater pressure, the material will transition from elastic wear to plastic wear. Rubber is classified as an elastic material. The sliding rubbing stage accounts for a significant proportion of abrasive grain extrusion. In this stage, the deformation of the workpiece surface can be fully recovered. When the grinding disk is tilted, the depth of the abrasive grit cuts from shallow to deep and then becomes shallow again, and the rubber material produces elastic deformation when the depth of cut is shallow, which results in the overall grinding depth and width being smaller than the theoretical grinding depth and width.

### 2.2. Geometry of Contact Area

**Figure 3:**
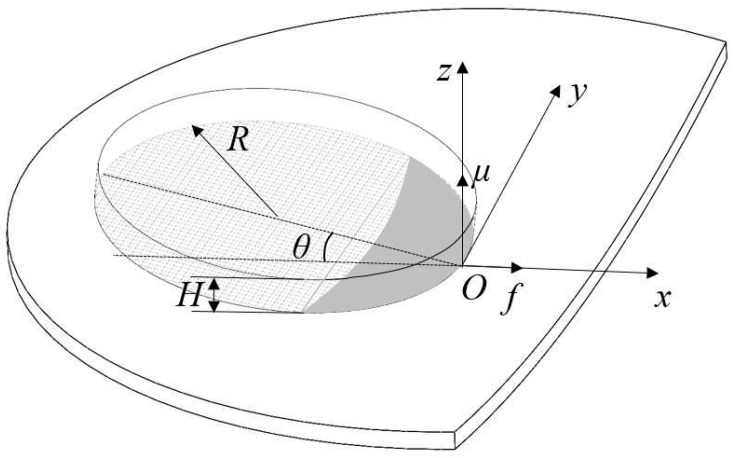
Schematic diagram of the contact area between the disc and the curved workpiece.

The radius of curvature of the grinding area of the rubber insulation layer is large. To explore the relationship between the grinding parameters and the contact force, the contact area between the flexible disc and the curved material to be ground is first modeled based on differential geometry theory, as illustrated in Figure 3. The spatial coordinate system is established as *O-xyz*; the grinding disc takes the contact point as the origin *O*, the feed direction is the same as the *x*-axis, the normal direction at the point *O* is the same as the *z*-axis, and the inclination angle between the grinding disc and the tangent plane of the material to be ground is *θ*; the thickness of the disc is *H*.

**Figure 4:**
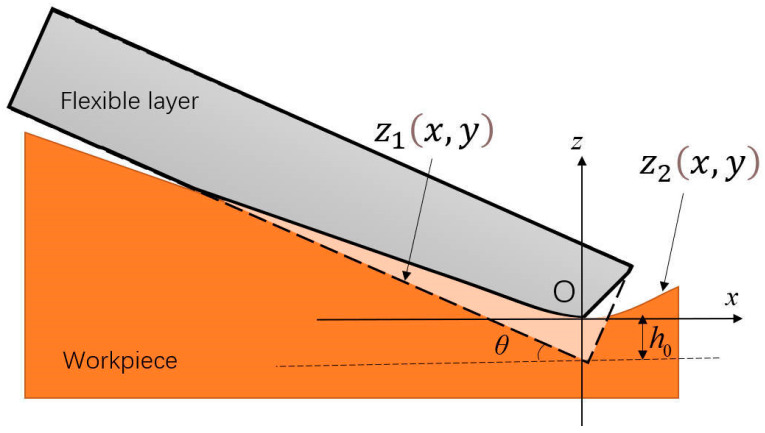
Area of elastic deformation of a disc in contact with a curved workpiece.

When a grinding disc is pressed into the material at an inclination angle *θ*, deformation occurs between the flexible layer of the grinding disc and the rubber surface (Figure 4). Since the deformation of the rubber in the contact area is significantly smaller than that of the flexible grinding disc, it can be disregarded. The undeformed disc base exhibits a circular shape, while its projection on the *x-y* plane forms an ellipse. The mathematical representation of the disc base is expressed as follows:

(1) $$\begin{array}{c} \left\{\begin{array}{c} z_{1}\left(x,y\right)=-x\cdot \tan\theta -h_{0}\\ {\left(\frac{x}{\cos\theta }+R\right)}^{2}+y^{2}\leq R^{2} \end{array}\right. \end{array}$$

In the equation, *θ* denotes the inclination angle between the disc base and the tangential plane of the material surface, while *R* represents the disc radius.

The rubber workpiece surface $z_{2}(x,y)$ is a surface of revolution. Given that the radius of curvature near the contact zone is significantly larger than the grinding disc radius *R*, the local region around the disc-material contact can be approximated as a quadratic surface, expressed as follows:

(2) $$z_{2}(x,y)=-\frac{1}{2}[xy]\left(\begin{array}{cc} a & c\\ c & b \end{array}\right)[xy{]}^{T}=-\frac{a}{2}x^{2}-\frac{b}{2}y^{2}-cxy$$

**Figure 5:**
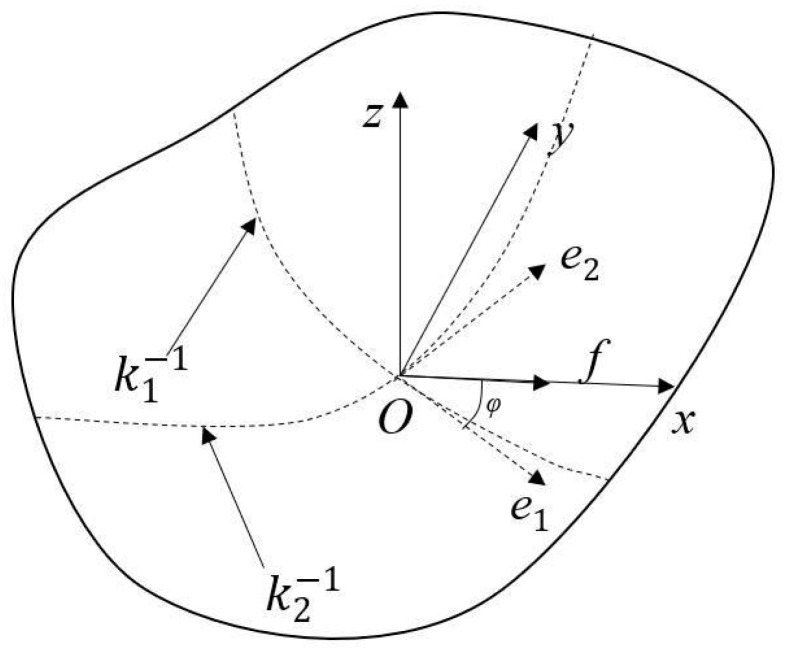
Schematic of a free-form workpiece surface.

In Equation (2), a,b,c are quadratic coefficients of surface curvature, $a=k_{1}\cos^{2}\varphi +k_{2}\sin^{2}\varphi$, $b=k_{1}\sin^{2}\varphi +k_{2}\cos^{2}\varphi$, $c=(k_{2}-k_{1})\sin\varphi \cos\varphi$. A schematic representation of the surface of a free-form workpiece is shown in Figure 5. $k_{1}$ is the maximum principal curvature, $k_{2}$ is the minimum principal curvature, φ is the angle between the direction of maximum principal curvature e_1_, and the direction of feed *f*. When the surface is concave, the curvature is negative. Different shapes of the workpiece surface and different feed directions affect the final shape of the contact area.

$z\left(x,y\right)$ is the distance between the bottom surface of the undeformed disc and the surface of the workpiece relative to the vertical along the *z*-axis, expressed as follows:

(3) $$z\left(x,y\right)=z_{1}-z_{2}=\frac{a}{2}x^{2}+\frac{b}{2}y^{2}+cxy-x\cdot \tan\theta -h_{0}$$

**Figure 6:**
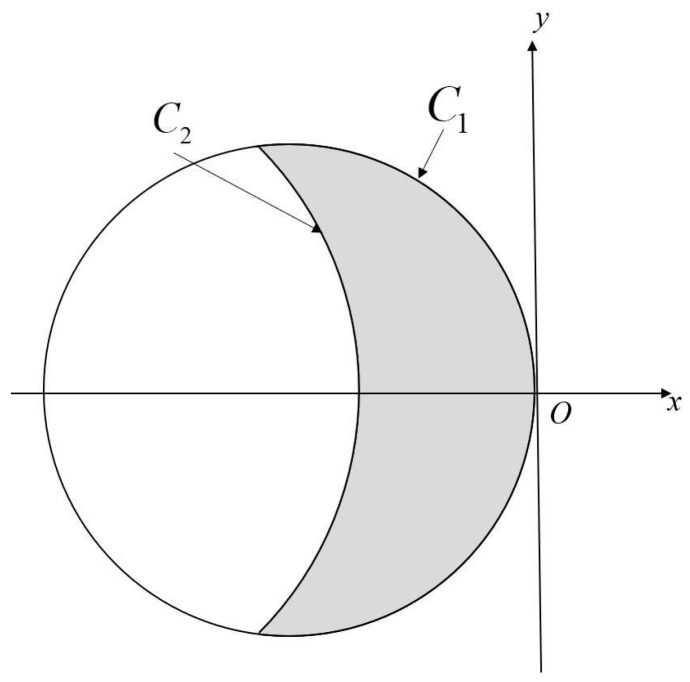
Schematic of the contact area.

The contact area between the bottom surface of the disc and the surface of the workpiece $z\left(x,y\right)$ ≤ 0, so the projection area of the contact area in the tangent plane of the contact point is a shaded area surrounded by two curves (Figure 6), where the curves *C*_1_ and *C*_2_ are expressed as follows:

(4) $$\begin{array}{c} C_{1}:\frac{x^{2}}{{cos}^{2}\theta }+2R\frac{x}{cos\theta }+y^{2}\leq 0\\ C_{2}:\frac{a}{2}x^{2}+\frac{b}{2}y^{2}+cxy-xtan\theta -h_{0}\leq 0 \end{array}$$

### 2.3. Modeling of Material Removal

In the field of material removal, similar to polishing and grinding rubber materials, a large number of studies have used the Preston equation for modeling and analysis as follows:

(5) $$\frac{dh}{dt}=k_{p}pv$$

In Equation (5), $\frac{dh}{dt}$ is the depth of material removal per unit time for a given trace, $k_{p}$ is the Preston coefficient, p is the pressure at the point of contact during grinding, and v is the relative speed of movement at the point of contact during grinding.

The Hertzian contact model is widely used. However, in this study, the grinding disc and workpiece maintain a specific inclination angle *θ*. As analyzed in the previous section, the contact zone deviates from an elliptical shape and exhibits a larger area. Under such tilted grinding conditions, the Hertzian contact model becomes inadequate. Therefore, we calculate the contact force based on the static elasticity theory by simplifying the elastic deformation in the contact region into multiple tiny springs, and the contact pressure at each point is calculated by the elastic strain at that point through, as follows:

(6) $$p=\bar{E}\delta^{\alpha }=\bar{E}(\frac{\left|z(x,y)\right|\cos\theta }{H}{)}^{\alpha }$$

In Equation (6), α and $\bar{E}$ are the nonlinear power indices and elasticity coefficients. p is the contact pressure, δ is the strain, and H is the total thickness of the sponge layer.

When grinding, the grinding disc is rotated at an angular speed ω and fed in a certain direction at a speed $v_{1}$ without stopping. The relative movement speed $v_{p}$ of each point on the disc with respect to the rubber material is the vector sum of the feed speed $v_{1}$ and the rotational linear speed $v_{R}$ of the point. Therefore, the relative movement speed *C* can be expressed as follows in Equation (7):

(7) $$v_{p}=\sqrt{{v_{1}}^{2}+{v_{R}}^{2}-2v_{1}v_{R}\cos\alpha }=\sqrt{{v_{1}}^{2}+(\omega (x-R){)}^{2}-2v_{1}(\omega (x-R))\cos\alpha }$$

In Equation (7), α is the angle between the direction of feed speed $v_{1}$ and the direction of rotational linear speed $v_{R}$ at the point.

### 2.4. Model Discretization

During the feeding process of the grinding disc, different areas of the grinding disc along the feed path will grind the same point on the rubber material, and the total material removal depth of the rubber material at that point can be obtained by integrating the material removal depth of each point on the path of the grinding disc, which is expressed as *MRD*(*y*).

During the grinding process, it is evident that the grinding width is smaller than the contact area width, with elastic deformation predominantly occurring in specific regions. Under low pressure or velocity conditions, rubber undergoes elastic deformation where molecular chains maintain relatively ordered arrangements. During plastic deformation, molecular chains experience tilting, stretching, and fracture, requiring increased energy expenditure [26]. The energy consumption for a single abrasive particle cutting through the material can be calculated using the equation [27]:

(8) $$E=v_{p}F_{t}t=v_{p}\mu F_{n}t$$

In the above equation, $v_{p}$ is the relative moving speed, $F_{t}$ is the tangential force, $F_{n}$ is the normal force, and μ is the friction coefficient.

The corresponding power *W* expression is as follows:

(9) $$W=\frac{E}{t}=\mu v_{c}F$$

The power increases as the rubber material moves from elastic deformation into plastic wear. For defined rubber materials and grinding discs, when the power W is less than *C*, only slipping occurs; the elastic deformation can be restored and does not show material removal [28]. In the grinding process of rubber materials, this stage accounts for a great proportion and cannot be ignored. The actual grinding zone must satisfy W(x,y)−C≥0. Defining f(x,y) as the grinding intensity at a given point, its expression can be derived as follows:

(10) $$f(x,y)=\left\{\begin{array}{cc} W(x,y)-C & W(x,y)-C\geq 0\\ 0 & W(x,y)-C<0 \end{array}\right.$$

In the above equation, *C* represents the power at the beginning of plastic deformation, which is related to the parameters of the rubber material.

According to Equation (5), the curve function of the cross-section of the grind traces at feed time $t_{0}$ should be obtained as follows:

(11) $$MRD\left(y\right)=\int_{0}^{t_{0}}k_{p}\left(\nu_{p}\left(t,y\right)p\left(t,y\right)-C\right)dt=\int_{0}^{t_{0}}k_{p}f(t,y)dt$$

In Equation (11), *MRD*(*y*) is the curve function of the cross-section; $k_{p}$ is Preston’s coefficient; $\nu_{p}\left(t,y\right)$ is the velocity distribution function of the corresponding coordinate point; and $p\left(t,y\right)$ is the stress distribution function of the corresponding coordinate.

The relative velocity distribution and stress distribution are unchanged during the feeding of the grinding disc in the region near the contact point. Consequently, the time-dependent integral in Equation (5) can be reformulated as a spatial integral along the feed direction (*x*-axis).

(12) $$\frac{dx}{dt}=v_{1}\to dt=\frac{dx}{v_{1}}$$

At this point, the curve function of the grinding cross section can be expressed as follows:

(13) $$MRD(y)=\frac{1}{v_{1}}\int_{C_{2}(y)}^{C_{1}(y)}k_{p}\cdot f(x,y)dx$$

In the above equation, $\nu_{p}\left(t,y\right)$ and $p\left(t,y\right)$ are the relative velocity and stress distribution curves at the corresponding points on the working surface of the grinding disc, respectively.

**Figure 7:**
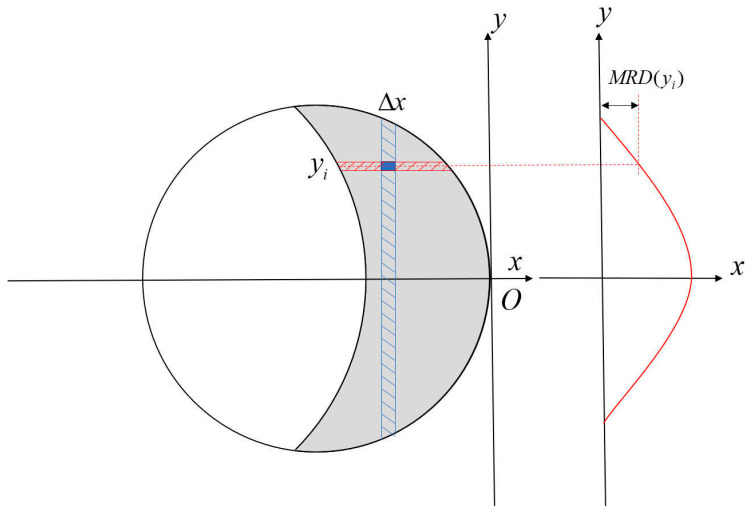
Schematic of the discretization method.

During the grinding process, the higher-order terms *x* and *y* of the boundaries *C*_1_ and *C*_2_ both appear on the left side of the equation, which produces complex elliptic integrals when integrating to obtain the depth of material removal, and an analytical solution cannot be found [23,24]. A spatiotemporal discretization method is adopted to decompose the continuous process into a linear superposition of discrete elements. The contact area is equally divided into N discrete segments along the feed direction of the grinding disc (*x*-axis). Within each segment, the cross-sectional direction (*y*-axis) is further discretized into M computational points, as shown in Figure 7.

Based on the grinding disc feed rate $v_{1}$, the time increment is converted to a spatial step:

(14) $$\frac{\Delta x}{\Delta t}=v_{1}\to \Delta t=\frac{\Delta x}{v_{1}}$$

Within the contact area, the coordinates of a discrete element are denoted as $\left(x_{i},y_{i}\right)$. The total material removal depth (MRD) at each computational point along the *y*-axis is expressed as MRD(y). The depth of material removal at a single discrete point can be expressed as follows:

(15) $$\Delta MRD(y_{i})=\frac{k_{p}}{v_{1}}f(x_{i},y_{i})\cdot \Delta x$$

Accumulation is performed for all discrete points, at which point the curve function of the ground cross-section can be expressed as follows:

(16) $$MRD(y_{i})\approx \frac{1}{v_{1}}\sum_{i=1}^{N}k_{p}\cdot f(x_{i},y_{i})\cdot \Delta x$$

In the above equation, $f(x_{i},y_{i})$ is the grinding strength at the corresponding point in the contact area of the grinding disc. Through this discretization method, the material removal depth $MRD(y_{i})$ at each point along the *y*-axis is determined, thereby establishing the cross-sectional profile of the grinding process.

**Figure 8:**
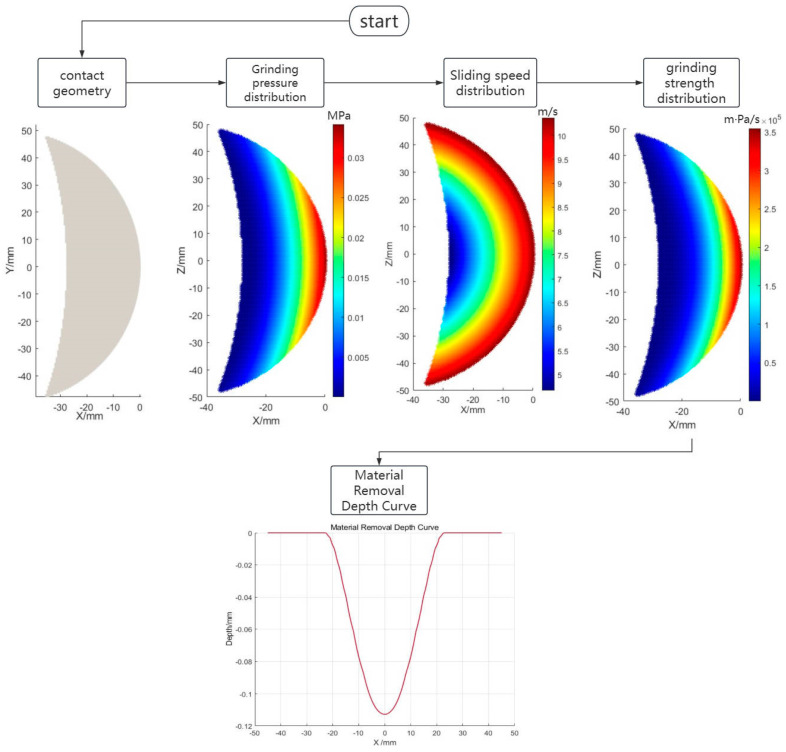
Flowchart for calculation of material removal depth curve.

The specific MRD curve acquisition flowchart is shown in Figure 8.

In the process of grinding, the measurement of the depth of downward pressure $h_{0}$ is challenging. Typically, the axial grinding pressure *F* is monitored during the grinding process. By multiplying the grinding pressure at each point with the corresponding area and cumulative to get the total grinding pressure of the contact surface, as shown in Equation (17):

(17) $$F=\sum_{i\in C_{1}\cap C_{2}}p(x_{i},y_{i})s_{i}$$

Because of the irregular shape of the contact area, it is not possible to give a direct relationship between the downward pressure depth $h_{0}$ and the grinding pressure *F*. The optimization method is used to obtain the correspondence between the downward pressure depth and the grinding pressure, as shown in Equation (18).

(18) $$h_{0}^{\prime }=\underset{h_{0}}{argmin}\left|F^{\prime }-\sum_{i\in C_{1}\cap C_{2}}p(x_{i},y_{i})s_{i}\right|$$

In Equation (18), *F*′ is the given target contact pressure, and the downward pressure depth $h_{0}$ is the optimization variable, different $h_{0}$ will give different grinding contact stress distributions and the final optimized maximum contact depth $h_{0}^{\prime }$.

## 3. Experimental Verification and Analysis

In order to verify the accuracy of the predictive model, grinding validation experiments are conducted under a range of conditions.

**Figure 9:**
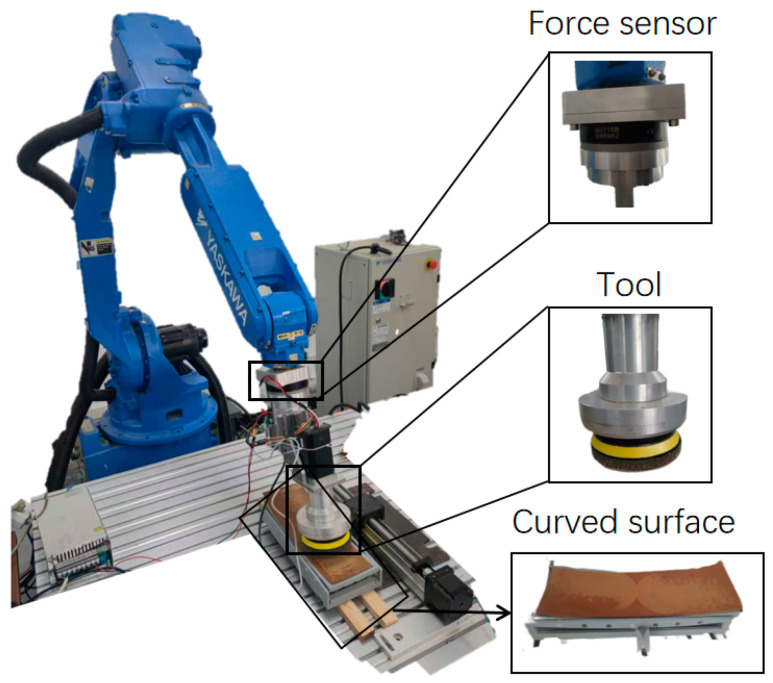
Experimental setup for surface grinding validation.

The experimental setup shown in Figure 9 consists of a six-axis robotic arm and a grinding device. The grinding device is mounted on the end of the MOTOMAN-HP20D robot arm of Yaskawa, Kitakyushu, Japan, and the grinding data are collected by the six-axis force sensor. The grinding depth information of the finished workpiece is measured using a 660 1-2 optical profiler of NanoFocus, Oberhausen, Germany, and the measurement results are processed in MATLAB R2023a to obtain the material removal depth information. The experimental material is EPDM rubber with a square geometry and a thickness of approximately 3 mm. In the grinding experiment, the rubber material is pasted on top of the curved base plate, and the grinding disc grinding device is controlled to press down to the set grinding pressure, after which the base plate is moved according to a predetermined trajectory, leaving a grinding mark on the grinding material.

The experimental subject primarily focuses on the rubber-based thermal insulation material inside the combustion chamber of a solid rocket motor. Its geometry is characterized by a curved surface, which shares similarities with the environmental contour described in Chapter 2. The experimental parameters for the grinding discs used are shown in Table 1.

The experiment is divided into two groups, one group tested the accuracy of the material removal model based on Preston’s theory by using two different down pressures of 15 N and 20 N and three different rotational speeds of 1600 rpm, 1800 rpm, and 2000 rpm to determine the accuracy of the material removal model. The other group is based on the easy measurement of the grinding pressure during the actual use, using a constant downward pressure of 20 N and a speed of 2000 rpm, two different inclination angles of 9° and 11°, and two different surface curvatures such as $k_{1}=1200\;mm^{-1},k_{2}=2000\;mm^{-1}$ and $k_{1}=1600\;mm^{-1},k_{2}=2000\;mm^{-1}$ to verify the accuracy of the contact area model.

### 3.1. Parametric Identification

In the process of pressure determination in the contact area, there are two unknown material parameters, the nonlinear power index α and the elasticity coefficient $\bar{E}$. When the tilt angle is certain, these two parameters can be fitted and identified by the downward pressure simulation. According to the material properties of the rubber and sponge to be treated, the Mooney-Rivlin and Yeoh intrinsic models are chosen to define the materials of the rubber insulation layer and the grinding disc sponge, respectively, and the specific material parameters are shown in Table 2 and Table 3.

**Figure 10:**
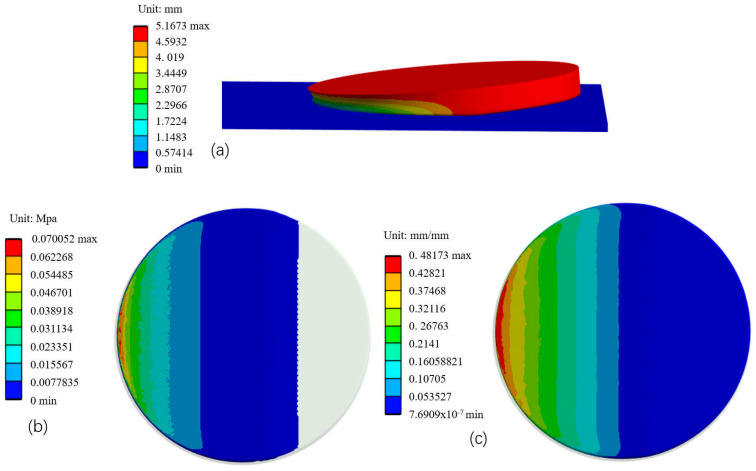
FEM method to test the interaction between the grinding disc and the workpiece. (**a**) Schematic of grinding disc deformation; (**b**) Contact Pressure Distribution; (**c**) Contact surface strain distribution.

**Figure 11:**
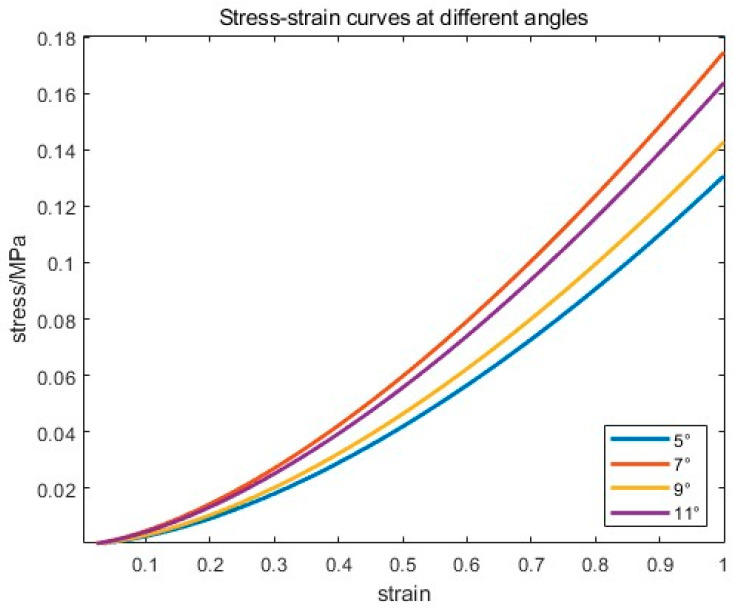
Measured contact stress versus strain curves.

In order to eliminate the influence of the workpiece, the downward pressure simulation is performed on a flat plate, as shown in Figure 10, and the midline pressure-strain curve obtained is shown in Figure 11.

The average nonlinear power index α=1.5930 and the elasticity coefficient $\bar{E}=0.1542$ are obtained by changing the simulation with different angles several times, we can get Equation (19):

(19) $$p=0.1542\delta^{1.593}$$

### 3.2. Experimental Verification

**Figure 12:**
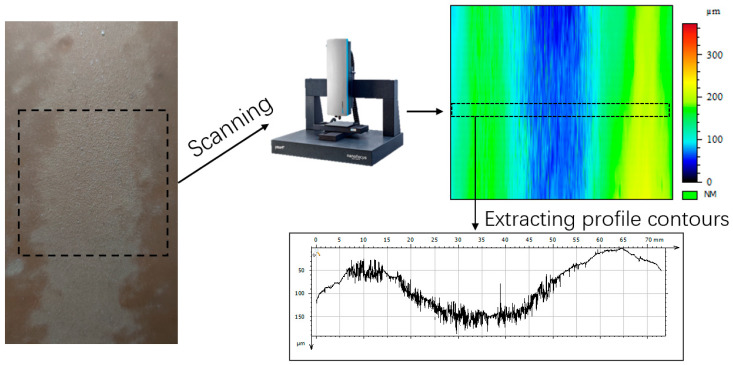
Surface contour extraction process for ground workpieces.

The existing rubber material grinding completed figure is as follows: the robotic arm in the grinding downward pressure process, there will be impact and grinding pressure fluctuations, thus leading to the problem of overcutting. For experimental analysis, a stable region of the grinding width is selected, and the removal contour is scanned perpendicular to the grinding disc’s movement direction. The actual grinding image and the scanned contour are presented in Figure 12.

The first step is to determine the required coefficients. Experiments are carried out on a flat workpiece with a grinding angle of 7° and a grinding pressure of 20 N. The material removal contour is measured, and the Preston coefficient *k_p_* between the grinding disc and the rubber insulation layer is calculated to be 1.04167 × 10^−8^, which is a constant that can be used to calculate the grinding path under other process parameters. The experimentally measured product C of pressure and speed at which plastic deformation begins is 2.989 × 10^5^ m·Pa/s.

**Figure 13:**
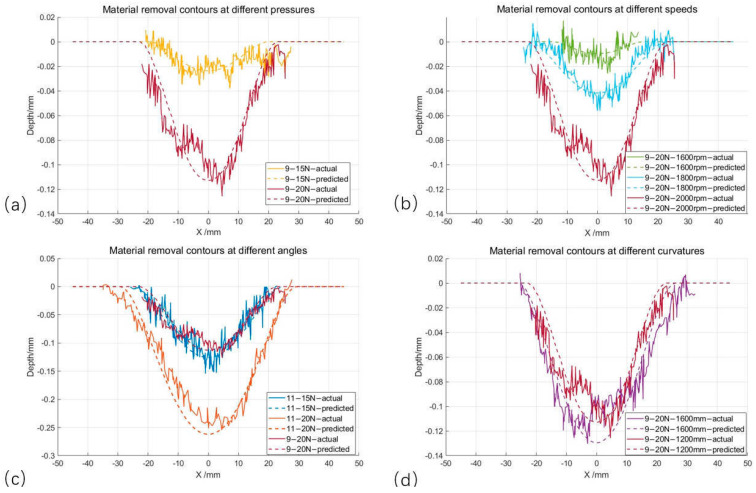
Comparison of grinding contour and predicted contour with different parameters. (**a**) Material removal contours at different pressures; (**b**) Material removal contours at different speeds; (**c**) Material removal contours at different angles; (**d**) Material removal contours at different curvatures.

By varying only one process parameter at a time, the shape of the grinding contour obtained is plotted against the model-fitted removal contour, as shown in Figure 13. The dashed line represents the removal contour calculated by the removal model, and the dotted line represents the removal cross-section contour obtained from the actual scanning. Table 4 shows the inaccuracies of the grinding contour and the predicted contour under different parameters. Under various working conditions, the model-fitted grinding contour has a better fitting effect than the actual grinding scanning contour, which can well reflect the material removal contour of the grinding process. This demonstrates that the proposed material removal model effectively calculates and analyzes the grinding contour. It is capable of obtaining information on grinding contour depth and width.

In addition, the experiments with different grinding parameters revealed the following pattern:

(1) Influence of downward pressure: Comparison of material removal contours with different pressures at the same angle in Figure 13a,c; when a larger amount of downward pressure is used, the deformation of the grinding disk is larger, and the contact width increases, but the deformation of the grinding disk increases, and the grinding pressure increases from 15 N to 20 N, increasing the contact pressure and increasing the depth and width of the material removal.
(2) The effect of grinding speed: (b) shows the use of different speeds under the material removal trajectory; the two grinding angles and grinding pressure are the same, the grinding speed from 1600 m increased to 2000 rpm, and the grinding depth from 0.0096 mm increased to 0.112 mm; the increase is more obvious. In the actual grinding, the fluctuation of the grinding rotational speed is greatly influenced by the grinding rotational speed, and it should be ensured that the grinding rotational speed is unchanged.
(3) Influence of inclination angle: Comparison of trajectories with different grinding angles under the same grinding pressure in (c); when the inclination angle increases, the contact area decreases, the pressure in the contact area increases, the amount of material removed increases, and the depth of material removed and the width of material removed increase.
(4) The effect of the shape of the Rubber insulation layer: In (d), two curvature radii are shown in the material removal contour; as the curvature radius decreases, the area of the grinding contact area decreases, the contact stress increases, and the actual grinding depth and grinding width both increase.

### 3.3. Influence of Shape Parameters

**Figure 14:**
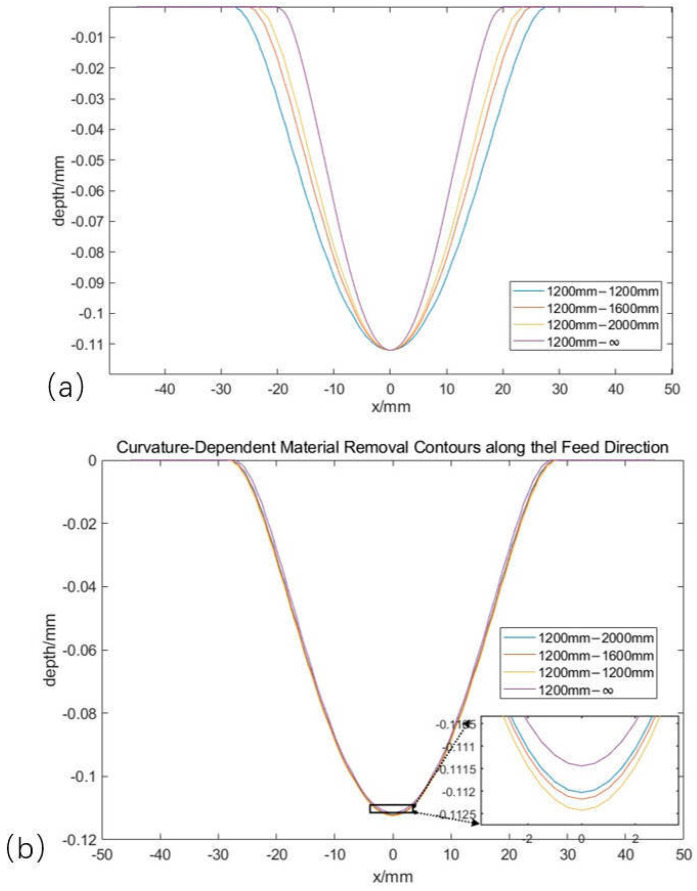
Predicted grinding contours with only different curvature directions. (**a**) Material removal contour with curvature change in perpendicular feed direction; (**b**) Material removal contour with curvature change along the feed direction.

During the actual grinding process, the main grinding parameters to be varied are the grinding pressure, the grinding angle, and the radius of curvature of the grinding material. The modeling analysis is carried out along the grinding feed direction and perpendicular to the grinding feed direction. The calculated material removal contour is shown in Figure 14. Where (a) represents the variation of the radius of curvature in the vertical grinding feeding direction, as the radius of curvature increases to infinity, the amount of grinding downward pressure remains unchanged, the grinding pressure decreases, the grinding depth remains almost unchanged, and the grinding width decreases from 55 mm to 41 mm. Figure 14b shows the variation of the radius of curvature along the grinding feed direction. When the radius of curvature increases to infinity, there is no obvious change in the contact stress at the edge of the grinding contact area where the rotational speed is high, and the rest of the area is less affected, which ultimately leads to no obvious change in the width of the grinding contour and the grinding depth. Analyzing the grinding contour of the grinding disc on different shapes of surfaces can help to complete the path planning of surface grinding.

**Figure 15:**
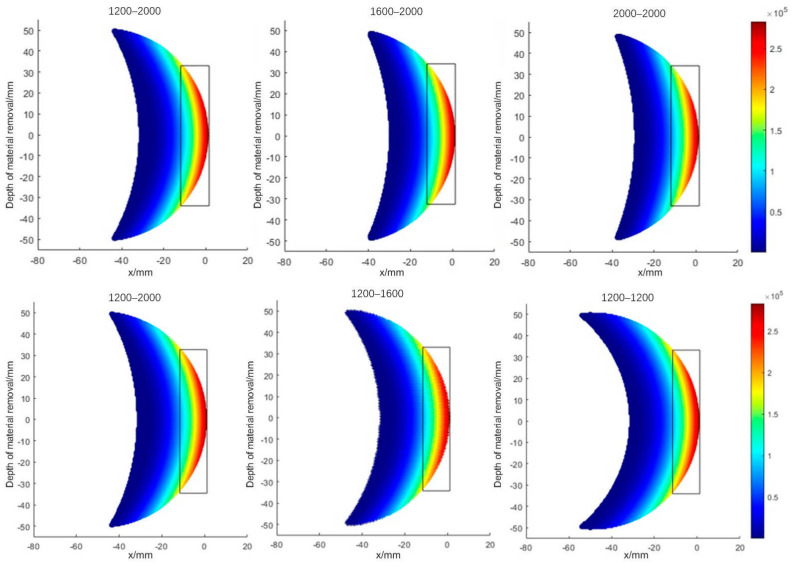
Plot of contact removal at different curvatures.

To find the reason behind the phenomenon, the distribution of contact removal at different curvatures of the workpiece is drawn, as shown in Figure 15. It can be observed that the size of the rectangular space on the graph is certain, and when the radius of curvature in the vertical grinding feed direction is changed, the curvature increases, and the area of larger grinding removal is concentrated at the tip of the grinding disc, so the grinding width decreases, but the maximum value of the grinding pressure does not increase, so there is no significant increase in the grinding depth. When the radius of curvature along the grinding feed direction is changed, the distribution area of material removal decreases with the increase in the radius of curvature, but there is no significant change in the area of the larger grinding pressure, no significant change in the maximum value of the grinding pressure, and no significant change in the final grinding depth and grinding width.

**Figure 16:**
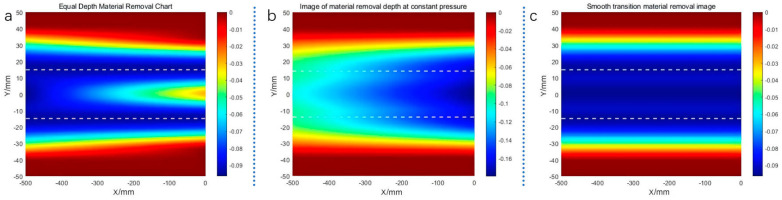
Distribution of material removal under different methods. (**a**) Equal Depth Material Removal Chart; (**b**) Image of material removal depth at constant pressure; (**c**) Smooth transition material removal image.

In actual surface grinding operations, two main methods exist: one is position control to ensure that the grinding depth is constant, meaning that the grinding path is parallel to the surface, and the other is constant force grinding, where the grinding pressure is controlled to be constant based on position control. When grinding free-form surfaces, the surface shape parameters will change before and after the same grinding path, and the grinding traces will change. Figure 16 below shows the free-form surface grinding traces. It can be seen that the change in curvature along the feed direction has less effect on the grinding depth and width, taking the radius of curvature to be unchanged at 2000 mm, and only changing the curvature in the perpendicular feed direction, the radius of curvature is changed from 1200 mm to 2000 mm. The grinding angle is set to 9°, the speed of grinding to be 2000 rpm, and the initial pressure of grinding to be 20 N. In Figure 16a, the amount of grinding downward pressure is constant, the grinding depth is constant, but the grinding width decreases with the increase in the curvature radius, which results in a shallow grinding depth at the larger curvature radius when multiple traces overlap; in Figure 16b, the grinding pressure is guaranteed to be constant, and the grinding depth and the grinding width increase with the increase in the curvature radius, which results in a less than uniform depth of the material removal; and in Figure 16c for the theoretical optimal material removal In Figure 16c, the theoretical optimal material removal is shown.

## 4. Conclusions

This study proposes a shallow material removal model for free-form surfaces of rubber materials, enabling the prediction of grinding trace cross-sectional profiles to optimize process parameters. The model introduces a pressure-velocity product term C to distinguish elastic deformation and plastic wear phases and discretizes complex contact zones into discrete points for calculating grinding profiles under varying pressures. Experiments under different parameters validated the model’s accuracy. This model can help to analyze the effect of different parameters on the contour of the depth of material removal, and the derived contour helps to analyze the effect of surface curvature on the grinding path, which will help the subsequent planning of overall grinding paths.

While material removal prediction is crucial, it cannot assess surface roughness, efficiency, or abrasive disc wear—factors influenced by path planning. Future work should integrate grinding path optimization and surface precision into the model for comprehensive prediction.

## Figures and Tables

**Table 1:** Experimental parameters for grinding discs.

Parametric Type	Parametric
Grinding disk type	100 mm diameter round grinding discs
Flexible layer thickness	12 mm
Grinding angle	9°, 11°
Grinding pressure	15 N, 20 N
RPM	1600 mm, 1800 rpm, 2000 rpm
Speed of movement	10 mm/s
Grit	80
Material type	EPDM
Workpiece curvature	$k_{1}=1200\;mm^{-1},k_{2}=2000\;mm^{-1}$ $k_{1}=1600\;mm^{-1},k_{2}=2000\;mm^{-1}$

**Table 2:** Parameters of the Mooney–Rivlin ontological model.

*C*_10_ (MPa)	*C*_01_ (MPa)
0.446	1.7849

**Table 3:** Parameters of the Yeoh ontological model.

*C*_10_ (MPa)	*C*_20_ (MPa)	*C*_30_ (MPa)	*D* (Pa-1)
0.01	0.007	0.0044	0.465

**Table 4:** Fitting accuracy of ground and predicted contours with different parameters.

Angle (°)	Pressure (N)	Speed (RPM)	Radius of Curvature (mm)	Width Relative Error	Depth Relative Error
9	15	2000	1200–2000	−5.915%	−2.315%
9	20	2000	1200–2000	−10.263%	−3.991%
9	20	2000	1600–2000	8.296%	−4.041%
9	20	1600	1200–2000	−11.070%	15.171%
9	20	1800	1200–2000	−8.150%	−9.186%
11	15	2000	1200–2000	−1.666%	−2.699%
11	20	2000	1200–2000	6.113%	1.827%

## Data Availability

The original contributions presented in this study are included in the article. Further inquiries can be directed to the corresponding author.
